# Functional Outcomes and Early Complications Following Volar Locking Plate Fixation of Unstable Distal Radius Fractures: A Minimum Two-Year Follow-Up Study

**DOI:** 10.7759/cureus.103133

**Published:** 2026-02-06

**Authors:** Saran Malisorn

**Affiliations:** 1 Orthopaedics, Naresuan University, Phitsanulok, THA

**Keywords:** carpal tunnel syndrome, complications, distal radius fracture, prwe, quickdash, volar locking plate

## Abstract

Background

Volar locking plate fixation has become the preferred operative treatment for unstable distal radius fractures because it allows stable fixation and early mobilization. However, long-term clinical outcomes and early complications in Asian populations treated in routine practice remain less well documented.

Methods

This retrospective cohort study included 40 consecutive adult patients with unstable distal radius fractures treated with volar locking plates by a single hand and wrist surgeon at a university hospital between January 2012 and December 2021. Patients met predefined radiographic criteria for instability and were followed for at least two years. Radiographic parameters (radial inclination, palmar tilt, and ulnar variance) were assessed preoperatively, immediately after surgery, at three months, and at ≥2 years. Wrist range of motion, grip strength, Quick Disabilities of the Arm, Shoulder, and Hand (QuickDASH), Patient-Rated Wrist Evaluation (PRWE), and pain visual analog scale (VAS) scores were recorded preoperatively, at two weeks, three months, and at ≥2 years. Early complications within three months (carpal tunnel syndrome, median nerve palmar branch injury, and extensor pollicis longus rupture) were retrieved from medical records. Paired t-tests were used to compare changes over time.

Results

A total of 40 patients were included (mean age, 55.4 years), of whom 26 (65.0%) were women. Radiographic alignment improved after fixation and was maintained at ≥2 years. Patient-reported outcomes (QuickDASH and PRWE), pain, range of motion, and grip strength improved progressively, with minimal disability and pain at long-term follow-up. Early symptoms consistent with carpal tunnel syndrome occurred in seven (17.5%) patients; no median nerve palmar branch injury, extensor pollicis longus rupture, or flexor pollicis longus rupture was identified.

Conclusions

In this single-surgeon retrospective cohort with a minimum two-year follow-up, volar locking plate fixation for unstable distal radius fractures achieved durable restoration of radiographic alignment and progressive improvement in wrist-related pain and function. Patient-reported outcomes, range of motion, and grip strength improved over time, and major complications were uncommon; early symptoms consistent with carpal tunnel syndrome occurred in seven (17.5%) patients, and no extensor pollicis longus or flexor pollicis longus rupture was identified. Overall, these findings support volar locking plate fixation as a reliable treatment option for unstable distal radius fractures in routine clinical practice.

## Introduction

Distal radius fractures (DRFs) are among the most common fractures of the upper extremity in adults, and their incidence is increasing, particularly among older women [1-5]. Several treatment options have been described for DRFs, including closed reduction and casting, external fixation, percutaneous pinning, dorsal plating, and volar locking plate fixation [6-8].

Over the past two decades, volar locking plate fixation has largely replaced dorsal plating and external fixation for unstable DRFs. Biomechanical and clinical studies have demonstrated that volar locking plates provide stable fixation with low complication rates and excellent functional outcomes [9-16]. Nevertheless, reported complication profiles vary, with particular concern regarding flexor tendon irritation, extensor pollicis longus (EPL) rupture, and median nerve complications.

Most published series originate from Western populations and focus on short- to mid-term outcomes, with relatively limited long-term functional and radiographic data [11-16]. A recent overview by Malisorn summarizes contemporary management strategies for fractures of the distal radius [17]. There remains comparatively limited long-term functional and radiographic evidence from Asian patients treated in routine clinical practice, particularly with standardized outcome measures and confidence intervals (CIs) that quantify the precision of estimates.

The purpose of this study was therefore to evaluate long-term clinical and radiographic outcomes and to describe early complications in patients with unstable DRFs treated with volar locking plates by a single hand surgeon at a Thai university hospital. It was hypothesized that volar locking plate fixation would provide durable restoration of alignment, excellent patient-reported outcomes at ≥2 years, and a low incidence of serious complications.

## Materials and methods

Study design and ethics

This was a single-center retrospective cohort study conducted at Naresuan University Hospital, Phitsanulok, Thailand. The study protocol, including the use of secondary data from medical records and hospital information systems, was approved by the Naresuan University Institutional Review Board (NU-IRB No. P3-0044/2566; COA No. 130/2023). The study was reviewed under the expedited pathway as secondary research; the requirement for written informed consent was waived because no direct patient contact or identifiable data collection occurred.

Patient selection

All consecutive adult patients who underwent volar locking plate fixation for unstable DRFs between 1 January 2012 and 31 December 2021 were screened. Eligible patients were required to have an unstable DRF resulting from low-energy trauma, classified as an extra-articular AO/OTA type A2.1, A2.2, A3.1, A3.2, or A3.3, or a simple articular fracture AO/OTA type C1.1, C1.2, C1.3, C2.1, or C2.2. Patients had to be at least 18 years of age, able to ambulate independently at the time of injury, and meet radiographic criteria for instability, defined as displacement greater than 2 mm, dorsal angulation greater than 10°, and/or the presence of an associated ulnar styloid fracture.

Patients were excluded if they had major carpal ligament injuries (such as scapholunate instability), open fractures or fractures requiring open reduction with bone grafting, ipsilateral proximal limb fractures other than an ulnar styloid fracture, or a prior wrist fracture in the same limb within the previous 12 months. Additional exclusion criteria included neurovascular injury or active infection at the operative site, ongoing radiotherapy, chemotherapy, or anticoagulation, metabolic bone disease other than osteoporosis, and any physical or mental condition that precluded reliable functional assessment.

Sample size was determined a priori based on the study objectives and statistical power. The primary outcome was defined as the change in Quick Disabilities of the Arm, Shoulder, and Hand (QuickDASH) score from baseline to ≥2 years. A two-sided paired t-test was planned (α=0.05; power=80%). Using a conservative standardized effect size of 0.60 (e.g., a 15-point clinically meaningful change with a standard deviation (SD) of 25 points), the minimum required sample size was 22 patients. To account for potential missing data and incomplete long-term follow-up, we targeted at least 30 cases. Because this was a retrospective study, all eligible patients during the study period were included (census sampling), yielding 40 cases. Given the observed magnitude of change in this cohort, the achieved power for the primary analysis was >99% [18].

Surgical technique

The hand and upper-extremity surgeon performed all procedures under general or regional anesthesia with tourniquet control.

A standard volar approach via the flexor carpi radialis (FCR) interval was used. The FCR tendon was retracted ulnarly, the radial artery was protected, the median nerve was protected, and the pronator quadratus was elevated in an L-shaped fashion to expose the distal radius. Fracture reduction was achieved under fluoroscopic guidance and temporarily stabilized with Kirschner wires. A precontoured volar locking plate was positioned on the volar surface of the distal radius proximal to the watershed line and secured with bicortical shaft screws and distal locking screws. Distal screws were intentionally selected 2 mm shorter than the measured depth to minimize dorsal penetration and extensor tendon irritation. Final fluoroscopic images confirmed fracture reduction, plate position, and screw length. The pronator quadratus and brachioradialis were repaired as anatomically as possible before closure.

For implants, fixation was performed using a volar locking plate system (Model VA; Bangkok Unitrade, Bangkok, Thailand) in all cases.

Postoperative management and follow-up

Postoperatively, the wrist was immobilized in a removable splint for comfort, while active finger motion was encouraged immediately. At approximately two weeks, sutures were removed, and wrist range-of-motion exercises were intensified. Clinical and radiographic follow-up was routinely performed at two weeks, 12 weeks, and at or beyond two years after surgery.

Relevant information regarding return to work and early complications was prospectively documented in clinic records. For this study, patients were contacted and invited to complete functional questionnaires (QuickDASH, Patient-Rated Wrist Evaluation (PRWE)) and a pain visual analog scale (VAS) at long-term follow-up (≥2 years).

Data collection

Data were extracted from the hospital information system and paper charts using a standardized case record form approved by the IRB. Collected variables included demographics (age, sex, side of injury, and AO/OTA classification); radiographic parameters (radial inclination, palmar tilt, and ulnar variance) recorded preoperatively, immediately postoperatively, at three months, and at ≥2 years; and clinical outcomes (wrist range of motion, grip strength, QuickDASH, PRWE, and pain VAS) recorded preoperatively, at two weeks, three months, and at ≥2 years. Early complications within three months (clinical carpal tunnel syndrome, median nerve palmar branch injury, and EPL tendon rupture) were also recorded.

Radiographic measurements (radial inclination, palmar tilt, and ulnar variance) were performed on standardized posteroanterior and lateral wrist radiographs using PACS measurement tools. Each parameter was measured twice by the same assessor at separate sittings, and the average value was used for analysis. Inter-observer reliability was not assessed and is acknowledged as a limitation.

Flexor tendon complications (including flexor pollicis longus (FPL) pathology) were assessed through review of follow-up clinic documentation for volar wrist pain, crepitus, triggering, weakness, or clinical suspicion of tenosynovitis. Any documented tendon rupture, advanced imaging (if performed), implant removal, or reoperation related to flexor tendons was recorded.

Patient-reported wrist-specific outcomes were assessed using the PRWE, a validated instrument developed to quantify wrist pain and disability [19,20]. When applicable, the validated Thai version (Th-PRWE) was used for Thai-speaking participants [21]. PRWE scoring followed standard instructions (0-100 total score; higher scores indicate worse pain/disability) [19,20,22,23]. Guidance on available PRWE user manuals and translations is provided by the Hand and Upper Limb Centre (St. Joseph's Health Care, London) outcome-measures resource [24]. Permission for non-commercial academic use and electronic administration of PRWE was confirmed by the instrument developer; documentation is provided in the Editor Review Files.

Statistical analysis

Continuous variables are presented as means, SDs, and 95% CIs. Categorical variables are presented as frequencies and percentages. Changes in continuous outcomes over time were assessed using paired t-tests. For paired categorical outcomes (carpal tunnel syndrome occurrence over time), McNemar’s exact test was used. Odds ratios (ORs) with 95% CIs were calculated for categorical comparisons, using a 0.5 continuity correction when zero cells were present. Statistical significance was set at p<0.05. Analyses were performed using IBM SPSS Statistics for Windows, Version 22 (Released 2013; IBM Corp., Armonk, New York).

## Results

Patient characteristics

A total of 40 patients met the inclusion criteria (Table 1). The mean age was 55.4 ± 11.3 years (range, 25-73). There were 26 (65.0%) women and 14 (35.0%) men. The right wrist was involved in 22 (55.0%) cases and the left in 18 (45.0%) cases. AO/OTA fracture patterns were predominantly B-type injuries, 17 (42.5%), and C-type injuries, 16 (40.0%), with the remaining 7 (17.5%) classified as A-type. All patients completed a minimum two-year follow-up for functional assessment.

**Table 1:** Demographic characteristics of the patients Values are presented as mean ± SD (95% CI) or n (%). No hypothesis testing was performed for descriptive baseline characteristics.

Characteristic	Value
Age (years)	55.4 ± 11.3 (51.8–58.9); range 25–73
Sex – Male	14 (35.0%)
Sex – Female	26 (65.0%)
Side – Right	22 (55.0%)
Side – Left	18 (45.0%)
AO/OTA – A2	1 (2.5%)
AO/OTA – A3	6 (15.0%)
AO/OTA – B2	8 (20.0%)
AO/OTA – B3	9 (22.5%)
AO/OTA – C1	4 (10.0%)
AO/OTA – C2	7 (17.5%)
AO/OTA – C3	5 (12.5%)

Radiographic outcomes

Radiographic alignment improved immediately after volar locking plate fixation and remained stable at the three-month and ≥2-year follow-up assessments (Table 2). Across all radiographic parameters, postoperative restoration was maintained without clinically meaningful deterioration over time, indicating durable maintenance of reduction.

**Table 2:** Radiographic parameters over time Values are presented as mean ± SD (95% CI); t-values and p-values were obtained using a two-sided paired t-test comparing preoperative and ≥2-year values.

Parameter	Preoperative mean ± SD (95% CI)	Immediate postoperative mean ± SD (95% CI)	3 months mean ± SD (95% CI)	≥2 years mean ± SD (95% CI)	t-value	p-value
Radial inclination (degrees)	12.1 ± 4.0 (10.8–13.4)	21.8 ± 1.0 (21.4–22.1)	21.8 ± 0.7 (21.5–22.0)	21.7 ± 0.7 (21.5–22.0)	16.410	<0.001
Palmar tilt (degrees)	-9.3 ± 6.0 (-11.3–-7.4)	6.4 ± 2.9 (5.5–7.4)	6.6 ± 2.8 (5.7–7.5)	6.7 ± 2.8 (5.8–7.5)	19.460	<0.001
Ulnar variance (mm)	2.1 ± 1.1 (1.7–2.4)	1.1 ± 0.5 (1.0–1.3)	1.2 ± 0.5 (1.0–1.3)	1.2 ± 0.4 (1.0–1.3)	5.902	<0.001

Functional outcomes

Patient-Reported Outcomes

Patient-reported outcomes improved substantially over time (Table 3). The largest gains occurred within three months, with continued improvement to ≥2 years. At final follow-up, most patients reported minimal pain and near-minimal disability. The magnitude of improvement in QuickDASH and PRWE far exceeded published minimal clinically important difference (MCID) thresholds, supporting clinical relevance at the group level [25,26].

**Table 3:** Functional outcomes, wrist range of motion, and grip strength over time Paired t-tests comparing preoperative and ≥2-year values for QuickDASH, PRWE, and VAS, and 2-week and ≥2-year values for range of motion and grip strength. QuickDASH: Quick Disabilities of the Arm, Shoulder, and Hand; PRWE: Patient-Rated Wrist Evaluation; VAS: Visual Analog Scale.

Outcome	Preoperative mean ± SD (95% CI)	2 weeks mean ± SD (95% CI)	3 months mean ± SD (95% CI)	≥2 years mean ± SD (95% CI)	t-value	p-value
QuickDASH score	87.2 ± 7.8 (84.7–89.7)	67.8 ± 7.5 (65.4–70.2)	26.7 ± 7.1 (24.4–29.0)	4.2 ± 2.6 (3.4–5.0)	76.311	<0.001
PRWE score	84.8 ± 6.5 (82.8–86.9)	41.4 ± 8.2 (38.8–44.0)	16.2 ± 5.9 (14.3–18.1)	3.2 ± 2.2 (2.5–3.9)	90.126	<0.001
Pain VAS (0–10)	9.2 ± 0.6 (9.0–9.4)	3.0 ± 0.6 (2.9–3.2)	0.8 ± 0.5 (0.7–0.9)	0.0 ± 0.0 (0.0–0.0)	96.977	<0.001
Wrist extension (degrees)	–	38.9 ± 5.7 (37.0–40.7)	53.5 ± 5.4 (51.8–55.2)	66.7 ± 5.7 (64.8–68.5)	30.846	<0.001
Wrist flexion (degrees)	–	37.1 ± 5.6 (35.4–38.9)	48.9 ± 8.3 (46.3–51.5)	63.4 ± 9.9 (60.2–66.5)	19.345	<0.001
Forearm supination (degrees)	–	47.5 ± 4.6 (46.0–48.9)	67.2 ± 7.3 (64.8–69.5)	81.4 ± 3.8 (80.2–82.6)	50.368	<0.001
Forearm pronation (degrees)	–	40.9 ± 4.0 (39.7–42.2)	57.9 ± 9.2 (54.9–60.8)	75.3 ± 4.4 (73.9–76.8)	51.626	<0.001
Grip strength (kg)	–	5.5 ± 0.6 (5.3–5.7)	9.8 ± 1.4 (9.3–10.2)	12.9 ± 2.9 (11.9–13.8)	17.652	<0.001

Range of Motion and Grip Strength

Wrist range of motion and grip strength improved progressively from the early postoperative period to ≥2 years (Table 3). Flexion-extension and forearm rotation demonstrated pronounced recovery by three months and approached near-normal values by long-term follow-up. Grip strength also increased over time, consistent with ongoing functional restoration.

Early complications

**Figure 1:**
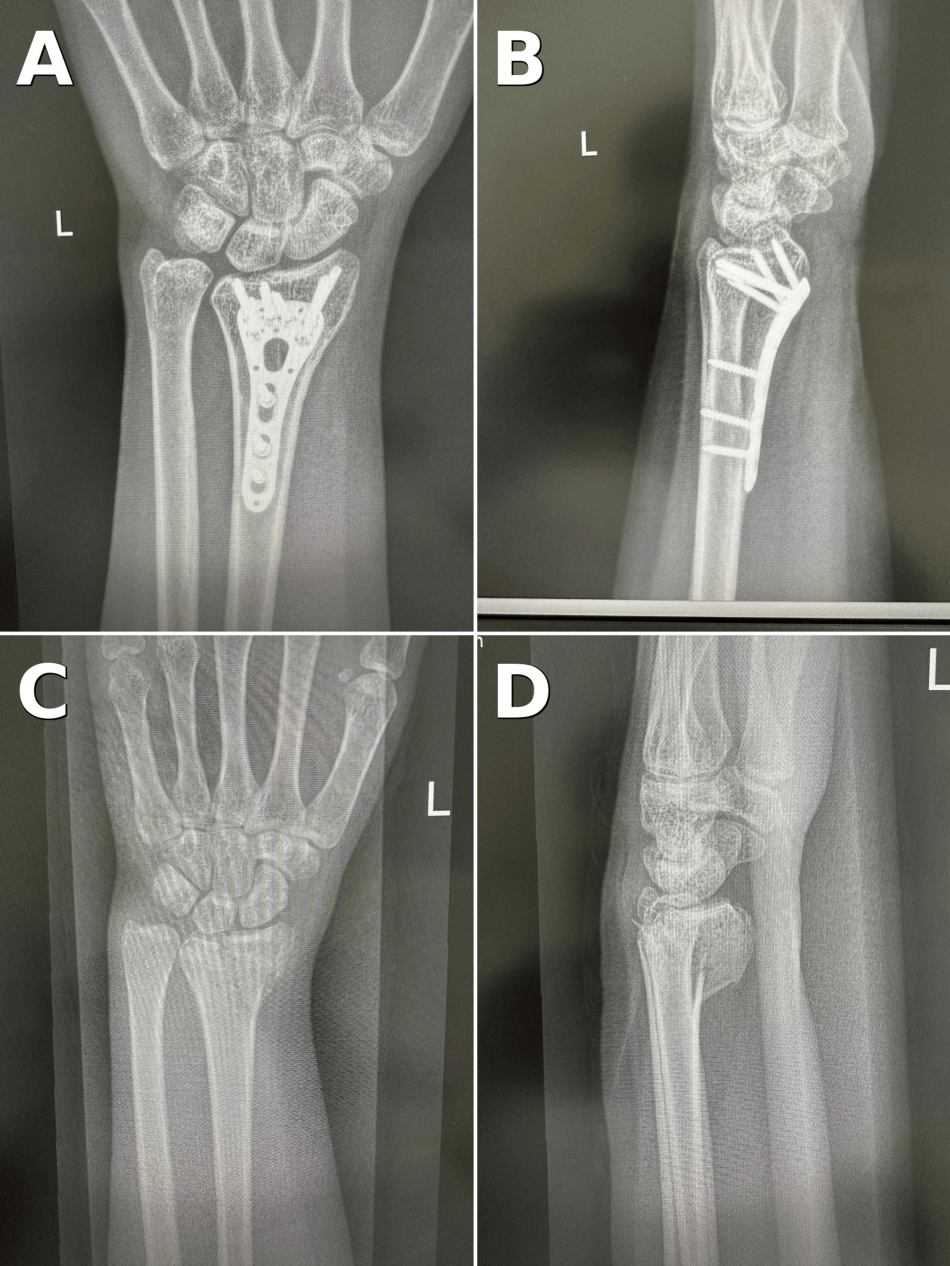
Pre- and postoperative radiographs of an unstable distal radius fracture treated with volar locking plate fixation (A, B) Immediate postoperative posteroanterior and lateral views of the left wrist showing restored radial height, radial inclination, and volar tilt with a volar locking plate positioned proximal to the watershed line. (C, D) Preoperative posteroanterior and lateral views demonstrating an extra-articular, dorsally displaced distal radius fracture with metaphyseal comminution and positive ulnar variance.

Early symptoms consistent with carpal tunnel syndrome were documented in 7 (17.5%) patients at the two-week visit (Table 4). No cases of median nerve palmar branch injury, EPL rupture, or FPL rupture were recorded. No additional carpal tunnel syndrome presentations were documented at three months or ≥2 years (Figure 1).

**Table 4:** Complications within the first three months and at ≥2 years Values are presented as the number of patients (%). ORs and 95% CIs were calculated using a 0.5 continuity correction due to zero cells. McNemar’s exact test was used for paired proportions (2 weeks vs. 3 months) where applicable. Flexor pollicis longus (FPL) rupture was assessed from follow-up clinical documentation; no cases were identified. OR: Odds Ratio; CI: Confidence Interval.

Complication	2 weeks (n=40)	3 months (n=40)	≥2 years (n=40)	OR	95% CI	χ² (McNemar)	p-value
Carpal tunnel syndrome	7 (17.5%)	0 (0.0%)	0 (0.0%)	15.0	0.86–262.9	5.143	0.016
Median nerve palmar branch injury	0 (0.0%)	0 (0.0%)	0 (0.0%)	NA	NA	NA	NA
Extensor pollicis longus rupture	0 (0.0%)	0 (0.0%)	0 (0.0%)	NA	NA	NA	NA
Flexor pollicis longus rupture	0 (0.0%)	0 (0.0%)	0 (0.0%)	NA	NA	NA	NA

## Discussion

In this series of 40 adult patients with unstable DRFs treated with volar locking plates by a single hand and wrist surgeon, there was reliable restoration and long-term maintenance of radiographic alignment, dramatic improvements in patient-reported outcomes, substantial gains in wrist motion and grip strength, and a low rate of significant complications.

The present results are consistent with previous studies reporting favorable outcomes after volar locking plate fixation [11-16]. The improvements in QuickDASH and PRWE scores, coupled with the narrow 95% CIs at ≥2 years, indicate substantial, clinically significant functional recovery. These findings extend prior research by offering long-term data from an Asian cohort and by clearly presenting 95% CIs around the estimates, aiding interpretation and facilitating comparison with other studies. The validated Thai version of the PRWE has been shown to be reliable and responsive in patients with DRFs [21].

Beyond statistical significance, the magnitude of improvement observed in this cohort suggests clinically meaningful recovery over time. Radiographic alignment was restored immediately after fixation and maintained through ≥2 years, supporting the stability of volar locked constructs. Similarly, patient-reported outcomes (QuickDASH and PRWE) improved substantially, with near-minimal disability at long-term follow-up. These findings are consistent with previously reported clinical series on volar locking plate fixation, which demonstrate reliable maintenance of reduction and favorable functional recovery [11-16]. Reporting 95% CIs alongside mean estimates provides additional transparency regarding the precision of outcomes in routine clinical practice.

Early symptoms consistent with carpal tunnel syndrome were noted in 7 (17.5%) patients, which is higher than in some reports but may reflect systematic documentation at the early postoperative visit when swelling is maximal. Importantly, these symptoms did not translate into persistent deficits at three months or ≥2 years, and no carpal tunnel syndrome-related reoperations were recorded.

No EPL ruptures were observed in this cohort, which contrasts with some previous reports of tendon ruptures after volar plating [12,15]. The absence of tendon complications in our series is likely due to careful selection of screw length and meticulous plate positioning proximal to the watershed line. No FPL rupture was identified; however, subclinical flexor tendinopathy may be under-ascertained in retrospective records when imaging is not routinely performed.

Strengths and limitations

This study has several strengths. All operations were performed by a single fellowship-trained hand surgeon using a consistent technique and postoperative protocol. All patients had at least two years of follow-up and a comprehensive assessment of radiographic and functional outcomes. The use of standardized measures (QuickDASH, PRWE, VAS) and the reporting of mean ± SD with 95% CI allow a more precise understanding of the magnitude and variability of treatment effects.

The study also has limitations. The retrospective design relies on the accuracy and completeness of existing records. This was a single-center cohort without a control group, which limits comparative conclusions. Although adequate to detect significant within-patient changes, the sample size restricts subgroup analyses. Because contralateral wrist measurements were not routinely recorded, motion and strength could not be expressed as a percentage of the unaffected side. Finally, the carpal tunnel syndrome diagnosis was based primarily on clinical assessment, with no systematic electrodiagnostic testing.

Clinical implications and future research

This study confirms that volar locking plate fixation can reliably restore and maintain radiographic alignment across a wide range of AO/OTA fracture patterns, helping surgeons treat unstable DRFs. Patients can expect statistically significant improvements in pain and function, with excellent long-term outcomes. Careful attention to plate position and screw length may reduce the risk of tendon problems. Early screening for carpal tunnel syndrome symptoms is warranted.

## Conclusions

Volar locking plate fixation for unstable DRFs in this single-surgeon series resulted in excellent restoration and durable maintenance of radiographic parameters, large and statistically significant improvements in QuickDASH, PRWE, pain, range of motion, and grip strength at ≥2 years, and a low incidence of significant complications. These findings support volar locking plate fixation as a safe and effective standard of care for unstable DRFs in adult patients.

## References

[1] Court-Brown CM, Caesar B (2006). Epidemiology of adult fractures: a review. Injury.

[2] Johansen A, Evans RJ, Stone MD (1997). Fracture incidence in England and Wales: a study based on the population of Cardiff. Injury.

[3] van Staa TP, Dennison EM, Leufkens HG, Cooper C (2001). Epidemiology of fractures in England and Wales. Bone.

[4] Thompson PW, Taylor J, Dawson A (2004). The annual incidence and seasonal variation of fractures of the distal radius in men and women over 25 years in Dorset, UK. Injury.

[5] Marquez MA, Melton LJ 3rd, Muhs JM (2001). Bone density in an immigrant population from Southeast Asia. Osteoporos Int.

[6] Kreder HJ, Agel J, McKee MD (2006). A randomized, controlled trial of distal radius fractures with metaphyseal displacement but without joint incongruity: closed reduction and casting versus external fixation. J Orthop Trauma.

[7] Rozental TD, Blazar PE, Franko OI (2009). Functional outcomes for unstable distal radial fractures treated with open reduction and internal fixation or closed reduction and percutaneous fixation: a prospective randomized trial. J Bone Joint Surg Am.

[8] Koval KJ, Harrast JJ, Anglen JO, Weinstein JN (2008). Fractures of the distal part of the radius: the evolution of practice over time. J Bone Joint Surg Am.

[9] Levin SM, Nelson CO, Botts JD (2008). Biomechanical evaluation of volar locking plates for distal radius fractures. Hand (N Y).

[10] Kandemir U, Matityahu A, Desai R, Puttlitz C (2008). Does a volar locking plate provide equivalent stability as a dorsal nonlocking plate in a dorsally comminuted distal radius fracture? A biomechanical study. J Orthop Trauma.

[11] Chung KC, Watt AJ, Kotsis SV (2006). Treatment of unstable distal radial fractures with the volar locking plating system. J Bone Joint Surg Am.

[12] Rozental TD, Blazar PE (2006). Functional outcome and complications after volar plating for dorsally displaced, unstable fractures of the distal radius. J Hand Surg Am.

[13] Chung KC, Squitieri L, Kim HM (2008). Comparative outcomes using the volar locking plating system for distal radius fractures in young adults and those older than 60 years. J Hand Surg Am.

[14] Jupiter JB, Marent-Huber M, LCP Study Group (2009). Operative management of distal radial fractures with 2.4-mm locking plates: a multicenter prospective case series. J Bone Joint Surg Am.

[15] Lattmann T, Meier C, Dietrich M, Forberger J, Platz A (2011). Results of volar locking plate osteosynthesis for distal radial fractures. J Trauma.

[16] Kwan K, Lau TW, Leung F (2011). Operative treatment of distal radial fractures with a locking plate system: a prospective study. Int Orthop.

[17] Malisorn S (2022). Fracture of distal end radius management: an overview. Int J Health Sci (Qassim).

[18] Hosokawa T, Tajika T, Suto M, Chikuda H (2020). Relationship between hand dominance and treatment outcomes for distal radius fractures in the elderly in the short-term. J Hand Surg Glob Online.

[19] MacDermid JC (1996). Development of a scale for patient rating of wrist pain and disability. J Hand Ther.

[20] MacDermid JC, Turgeon T, Richards RS, Beadle M, Roth JH (1998). Patient rating of wrist pain and disability: a reliable and valid measurement tool. J Orthop Trauma.

[21] Laohaprasitiporn P, Monteerarat Y, Jaderojananont W, Limthongthang R, Vathana T (2021). Validity, reliability and responsiveness of the Thai version of Patient-Rated Wrist Evaluation (Th-PRWE) in distal radius fracture patients. Siriraj Med J.

[22] MacDermid JC (2019). The PRWE/PRWHE update. J Hand Ther.

[23] Shafiee E, MacDermid J, Farzad M, Karbalaei M (2022). A systematic review and meta-analysis of Patient-Rated Wrist (and Hand) Evaluation (PRWE/PRWHE) measurement properties, translation, and/ or cross-cultural adaptation. Disabil Rehabil.

[24] St. Joseph’s Health Care London (2026). St. Joseph’s Health Care London Outcome Measures (Wrist/Hand: PRWE user manual and translations). https://www.sjhc.london.on.ca/research/outcome-measures.

[25] Sorensen AA, Howard D, Tan WH, Ketchersid J, Calfee RP (2013). Minimal clinically important differences of 3 patient-rated outcomes instruments. J Hand Surg Am.

[26] Walenkamp MM, de Muinck Keizer RJ, Goslings JC, Vos LM, Rosenwasser MP, Schep NW (2015). The minimum clinically important difference of the Patient-Rated Wrist Evaluation score for patients with distal radius fractures. Clin Orthop Relat Res.

